# Trait Emotional Intelligence in Childhood: Factorial Structure of the TEIQue–Child Form (CF) and Child Short Form (CSF)

**DOI:** 10.3390/bs16040501

**Published:** 2026-03-27

**Authors:** Stella Mavroveli, Konstantinos V. Petrides, Maria-Jose Sanchez-Ruiz

**Affiliations:** 1 Department of Surgery and Cancer, Imperial College London, London SW7 2AZ, UK; 2 London Psychometric Laboratory, University College London, London WC1H 0AP, UK; k.petrides@ucl.ac.uk; 3 Department of Personality, Assessment, and Psychological Treatment, Universidad Nacional de Educación a Distancia (UNED), 28040 Madrid, Spain; mj.sanchezruiz@psi.uned.es

**Keywords:** trait emotional intelligence, TEIQue-CF, TEIQue-CSF, child development, factor analysis

## Abstract

This research examined the component structure of two child measures, the Trait Emotional Intelligence Questionnaire–Child Form (TEIQue-CF; 75 items) and its short form (TEIQue-CSF; 36 items), developed specifically for children aged 8 to 12 years. Study 1 analysed TEIQue-CF data using the nine facet scores from 720 UK primary school pupils in Years 3 through 6 using principal component analysis with parallel analysis for factor retention. Results supported a unifactorial solution in the total sample, with a single factor explaining 43.48% of the variance. Exploratory subgroup factor analyses (in boys and older children in Years 5 to 6) in Study 1 suggested a potentially interpretable bifactorial pattern, though parallel analysis did not support retaining the second factor. Study 2 examined the TEIQue-CSF in 1582 Year 6 pupils using parcel-level analysis. A clearer two-factor structure emerged, with Socioemotionality (Adaptability, Peer relations, Self-esteem, Emotion expression, Affective disposition, Emotion perception) and Emotion control (Impulse control, Emotion regulation, Self-motivation) explaining 53.7% of the variance. This structure replicated across gender subgroups. Taken together, the findings suggest a developmental trend in which trait EI shifts from a largely undifferentiated structure in middle childhood to a more differentiated two-factor organisation by the end of primary school. They support the use of global trait EI scores in younger children while indicating that differentiated assessment becomes appropriate as children approach adolescence.

## 1. Introduction

Trait emotional intelligence (trait EI) refers to a constellation of emotional perceptions assessed via questionnaires and rating scales (Petrides et al., 2007). The construct is conceptually aligned with the subjective nature of emotional life and taps information that is primarily available to the individual. Despite a mature adult literature (see Petrides et al., 2016 for a review), work with children, especially in middle and late childhood, has been comparatively sparse. A common impediment in such research is the tendency to apply successful adult measures lock, stock, and barrel to children without due regard for developmental constraints, which may obscure age-related differences in how children process emotions, construe, and report on their emotional selves (Mitchell et al., 2022; Nook et al., 2020).

Within the trait EI framework, the Trait Emotional Intelligence Questionnaire (TEIQue; Petrides, 2009) family provides a comprehensive operationalisation of emotion-related personality. For primary-aged children, two forms currently exist: the TEIQue–Child Form (TEIQue-CF) and the TEIQue–Child Short Form (TEIQue-CSF). These forms were developed specifically for ages 8 to 12 through a staged process that first delineated a child-appropriate sampling domain (see Table 1) and then generated and refined items at an accessible reading level.

Piloting included classroom comprehension checks with targeted wording revisions where necessary. Statements are rated on a 5-point scale and provide broad coverage of children’s emotion-related personality. The final TEIQue-CF comprises 75 items across nine facets, written at a primary-appropriate reading level. The TEIQue-CSF is a 36-item short form with 3 to 4 items per facet, designed to yield a reliable global trait EI score. Because this short form targets a single overall score, facet-level interpretations are not appropriate. For structural analyses, we therefore work at the parcel level, creating nine parcels by aggregating the items representing each facet.

In adults, the TEIQue consistently yields four broad factors, namely Well-being, Self-control, Emotionality, and Sociability, and this structure has been replicated across cultures and samples (e.g., Aluja et al., 2016; Mikolajczak et al., 2007; Perez-Diaz & Petrides, 2021; Sanchez-Ruiz et al., 2021). By contrast, in middle childhood, self-perceptions are still relatively undifferentiated as compared to adolescence, in which they are more complex and integrated (e.g., Harter, 2012). As children move through the primary years, a richer emotion vocabulary, stronger perspective taking, and consistent feedback from teachers and peers help them distinguish feeling, expressing, and regulating emotions (Grosse et al., 2021; Sorlie et al., 2020). Relatedly, executive function and metacognitive monitoring shift from relatively unitary to more differentiated structures across late childhood and adolescence (Roebers, 2017), while reduced response biases and improved use of rating scales yield more elaborate facet and factor structures.

From a developmental perspective, children’s self-reports therefore shift from broad, global self-evaluations toward more differentiated and domain-specific self-knowledge as cognitive and metacognitive capacities mature (e.g., Harter, 2012; Roebers, 2017). Accordingly, differences in the dimensional structure of trait emotional intelligence self-reports across primary school years may reflect normative developmental differentiation in emotional self-concept.

The present research was designed to examine and evaluate the observed dimensional structure of the TEIQue-CF and TEIQue-CSF in middle and late childhood, specifically investigating whether children’s responses are best characterised by a single broad dimension or whether evidence of multidimensional differentiation emerges, particularly in older primary school cohorts. Some factor analytic work has already been conducted (Banjac et al., 2016; Beyazit et al., 2020), but the scientific literature still lacks easily accessible evidence regarding the internal dimensional structure of these instruments. While common factor models are often preferred for inferring latent structures, PCA was selected here as an initial exploratory technique to identify major patterns of variance in the data, consistent with prior dimensional analyses of trait EI measures and to facilitate comparison with existing literature. The manuscript comprises two complementary studies designed to clarify and make accessible the structural organisation of trait EI indicators across middle and late childhood.

## 2. Study 1

Study 1 evaluates the dimensionality of the full 75-item TEIQue-CF in a large community sample of UK primary school pupils in Years 3 through 6. The goal is to provide transparent, facet-level evidence on the breadth and coherence of children’s trait EI self-perceptions, clarifying how many latent dimensions are warranted in this age range and how these dimensions are organised.

To address this goal, we employed a rigorous exploratory approach to factor retention using principal component extraction with oblique rotation. Factor retention decisions were guided by multiple criteria, including Parallel Analysis (PA). PA generates eigenvalues from randomly simulated correlation matrices matched to the actual data’s dimensions based on the same number of variables and participants. Factors are retained when observed eigenvalues exceed their corresponding random eigenvalues, indicating genuine underlying structure rather than chance variation. We implemented PA using the Marley Watkins Monte Carlo PCA program (Watkins, 2000) with 1000 replications, applying the 95th percentile threshold (5% significance level) for comparison with random eigenvalues. We also explored potential developmental and gender differences by repeating the structure-finding steps across age and sex subgroups, enabling a targeted assessment of whether the emerging configuration was stable or showed early signs of differentiation. Missing data were minimal, <10%; therefore, to ensure data completeness, missing values were imputed using mean imputation.

### 2.1. Method

#### 2.1.1. Participants

Participants were 720 primary school children in Years 3 through 6 (343 boys, 372 girls, 5 unspecified; *M* = 9.16 years, *SD* = 1.25). Boys had a mean age of 9.15 years (*SD* = 1.32) and girls had a mean age of 9.16 years *(SD* = 1.18). Participants were divided into younger (Years 3–4; *n* = 342, *M* age = 8.19, *SD* = 0.73) and older (Years 5–6; *n* = 331, *M* age = 10.26, *SD* = 0.70) age groups. All pupils attended schools within the Greater London area. The ethnic distribution was 27% White UK heritage, 19% White other, 17% White European, 7% Black African, 6% Bangladeshi, 5% Indian, 19% other ethnicities, and 1% not reported.

#### 2.1.2. Measure

The TEIQue-CF is a self-report inventory that provides nine facet scores and a global trait EI composite using brief first-person statements rated on a five-point response format. It is suitable for classroom administration and worded for primary pupils. In the present sample, internal consistency for the global score was 0.90, and facet reliabilities were in the expected range for short subscales (approximately 0.57 to 0.77), with somewhat higher coefficients in Years 5 and 6 than in Years 3 and 4. These indices support the use of the global score and provide acceptable facet-level precision for research purposes.

#### 2.1.3. Procedure

Schools in Greater London were invited by letter outlining the study aims and procedures, and interested schools provided institutional consent by returning a signed form. Participating schools then received detailed information describing administration and data handling. Before data collection, parents/guardians were informed about the study and provided consent in line with the process outlined within the ethical application. In each class, a teacher and a teaching assistant administered the questionnaires during a morning lesson before lunch, following a standard protocol. Pupils were informed that participation was voluntary, that they could withdraw at any point without penalty, and that they could leave any question unanswered. To protect confidentiality, pupils completed their questionnaires individually and sealed them in an envelope before handing them to the teacher. Ethical approval for this study was obtained by the Institute of Education Ethics Committee from the first author and was prepared in line with the ethical standards delineated by the British Psychological Society and the Declaration of Helsinki.

#### 2.1.4. Statistical Analyses and Factor Retention Criteria

Prior to conducting exploratory factor analysis, the suitability of the data was assessed. The Kaiser–Meyer–Olkin (KMO) measure of sampling adequacy was 0.87 (exceeding the acceptable threshold of 0.60), and Bartlett’s test of sphericity was significant, χ^2^(36) = 1853.27, *p* < 0.001, indicating that the correlation matrix was appropriate for factor analysis.

PCA was selected as an exploratory data-reduction technique to identify major sources of variance in the item set, consistent with its use in prior trait EI scale examinations and early dimensional work, while acknowledging that common factor analysis would be preferable for strictly latent-variable inference. Exploratory analyses were conducted using PCA followed by Direct Oblimin rotation with Kaiser Normalization since facets were expected to intercorrelate. Even though parallel analysis was the primary retention criterion, additional solutions were examined exploratorily for conceptual interpretability. Interpretations of non-retained structures remain descriptive and provisional. All analyses were performed in SPSS 29 and 31. Multiple criteria were employed to determine the appropriate number of factors to retain, following recommendations by Thompson and Daniel (1996): visual inspection of the scree plot, Kaiser’s eigenvalue-greater-than-one criterion (K > 1), and PA.

### 2.2. Results

#### 2.2.1. Score Distributions and Internal Consistencies

Descriptive statistics, item counts, internal consistencies, and intercorrelations for the nine TEIQue-CF facets are presented in Table 2. Score distributions appeared relatively normal (<1.5; Stevens, 2002), although the Kolmogorov–Smirnov (K-S) test indicated departures from normality for most facets, with six of nine showing statistically significant deviations (*p*s < 0.05) because of the large sample size (*N* = 720). Three facets (Emotion expression, Emotion perception, and Impulse control) did not significantly depart from normality. In addition, normality was also achieved for the global trait EI score (K-S (720) = 0.021, *p* > 0.05).

**Figure 1:**
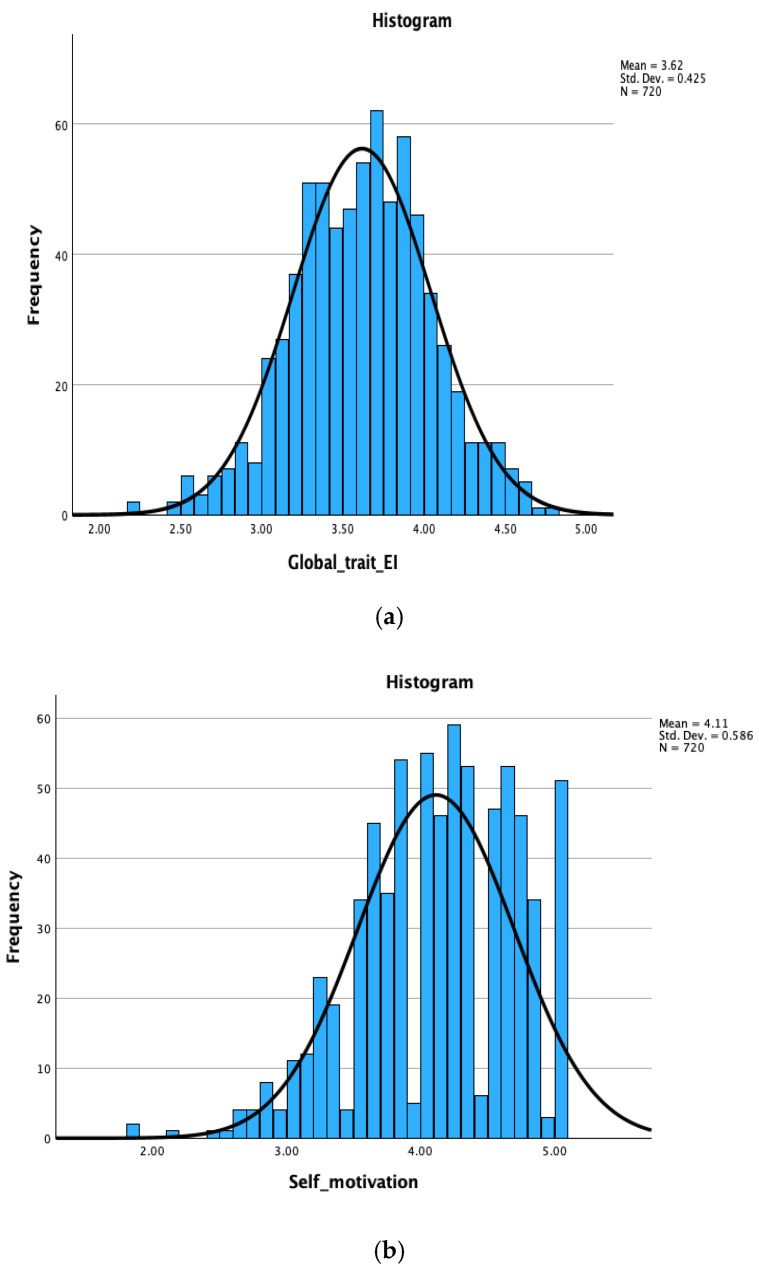
(**a**). Frequency Distribution for Global Trait EI (*N* = 720). (**b**). Frequency Distribution for Self-motivation (*N* = 720).

Example distributions are presented in Figure 1a,b below. As expected, for some facets (e.g., Affective disposition, Peer relations, Self-esteem, and Self-motivation), the data were negatively skewed (see Table 2). Children’s responses can be positively biased (e.g., Thomaes et al., 2017), and we considered these results normal. For example, the non-normal distribution of the Self-motivation facet is unsurprising, given that the facet comprised items related to schoolwork (e.g., “I try to do my homework as well as I really can.”). The classroom context and the presence of the teacher might also have influenced children’s responses.

In this sample, the internal consistencies of the nine facets were satisfactory, with the exception of Emotion perception (α = 0.57), Adaptability (α = 0.59), and Emotion expression (α = 0.59), which were slightly below recommended minimum values (see Table 2). These results were anticipated due to the small number of items in each subscale (fewer than 10) and the fact that each facet is called upon to provide coverage of a broad underlying theoretical construct. Repetition and rewording of the items (paraphrasing) were avoided because the respondents were children, and long or tedious questionnaires are not advisable with this age group. According to Loewenthal (2001), a lower reliability index (around 0.60) can be acceptable when the number of items is fewer than 10, and this approach is preferable to using long and repetitive scales.

Age-specific reliability analyses revealed that the younger children responded less consistently on some of the TEIQue-CF facets compared to the older children, while the internal consistency of the Emotion perception facet showed a low alpha (α < 0.60) in both age groups. For younger children, four facets had Cronbach’s alpha values below 0.60 (Adaptability, Emotion expression, Emotion perception, and Emotion regulation; see Table 3).

#### 2.2.2. Factor Analysis

##### Total Sample

**Figure 2:**
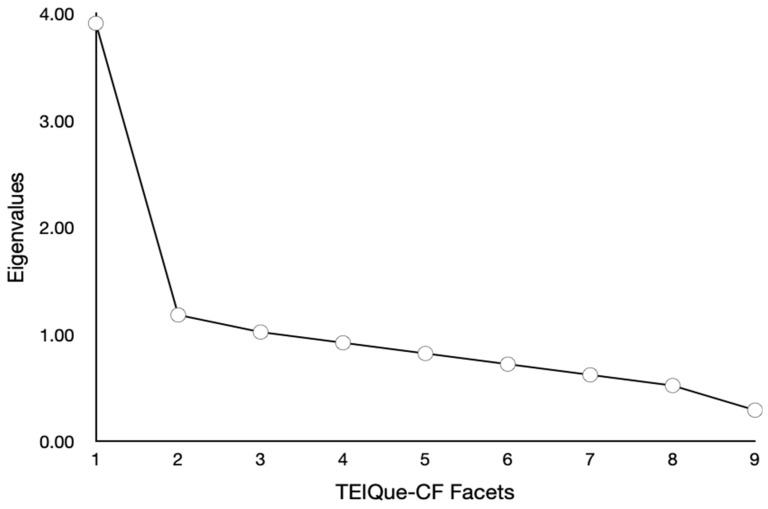
Scree Plot for PCA of the Nine Facets of the TEIQue-CF for the Total Sample (*N* = 720).

The nine TEIQue-CF facets were subjected to a PCA in the total sample (*N* = 720; *M* age = 9.16, *SD* = 1.25). Based on the scree plot (see Figure 2) and Kaiser’s K > 1 criterion, a unifactorial solution appeared to provide the best fit to the data. Parallel Analysis (9 variables, 720 participants, 1000 replications) confirmed this decision: the first observed eigenvalue (3.91) exceeded the 95th percentile random eigenvalue (1.22), while subsequent eigenvalues did not. The single extracted factor had an eigenvalue of 3.91 and explained 43.48% of the total variance.

All nine facets demonstrated adequate factor loadings (range: 0.54 to 0.76; all loadings > 0.40) on the extracted factor. Communalities ranged from 0.30 to 0.59 (*M* = 0.44). This single factor can be interpreted as representing a generalized (positive or negative) view of the emotion-related aspects of the self. This finding is consistent with the initial hypothesis that the conceptualization of trait EI in children would differ meaningfully from that in adults, particularly in exhibiting a less differentiated factor structure.

#### 2.2.3. Subgroup Analyses by Gender

*Gender differences*. The data were subsequently factor analyzed separately by gender. For girls (*n* = 372; *M* age = 9.16, *SD* = 1.18), the KMO was 0.88, and Bartlett’s test was significant, χ^2^(36) = 1027.65, *p* < 0.001. The scree plot and Kaiser’s K > 1 criterion both suggested a one-factor solution. Parallel Analysis (9 variables, 372 participants, 1000 replications) confirmed this decision: the first observed eigenvalue (4.05) exceeded the 95th percentile random eigenvalue (1.31), while the second observed eigenvalue (0.92) did not. The single extracted factor had an eigenvalue of 4.05 and explained 44.96% of the variance. All nine facets demonstrated adequate factor loadings (range: 0.57 to 0.79; all loadings > 0.40) on the extracted factor. Communalities ranged from 0.32 to 0.62 (*M* = 0.45).

For boys (*n* = 343; *M* age = 9.15, *SD* = 1.32), the KMO was 0.84, and Bartlett’s test was significant, χ^2^(36) = 834.34, *p* < 0.001. To determine the most appropriate factor solution, multiple criteria were employed. The scree plot suggested a two-factor solution. Applying Kaiser’s K > 1 criterion, two factors were extracted accounting for 53.06% of the total variance. However, Parallel Analysis (9 variables, 343 participants, 1000 replications) indicated only one factor should be retained: while the first observed eigenvalue (3.74) exceeded the 95th percentile random eigenvalue (1.33), the second observed eigenvalue (1.04) did not exceed the corresponding random eigenvalue (1.22), suggesting the two-factor solution may be artifactual.

Despite the PA recommendation for a one-factor solution, the two-factor structure was examined due to theoretical interest in potential emerging differentiation. The two-factor solution for boys was rotated using Direct Oblimin rotation with Kaiser Normalization. As shown in Table 4, the first factor (eigenvalue = 3.74, 41.56% variance) comprised six facets: Self-esteem (0.72), Peer relations (0.79), Adaptability (0.75), Emotion expression (0.58), Emotion perception (0.50), and Affective disposition (0.46). The second factor (eigenvalue = 1.04, 11.50% variance) comprised three facets: Impulse control (0.90), Emotion regulation (0.71), and Self-motivation (0.52). The first factor was labelled Socioemotionality, and the second factor was labelled Emotion control. The correlation between the two factors was 0.40.

#### 2.2.4. Subgroup Analyses by Age

For younger children (Years 3–4; *n* = 342; *M* age = 8.19, *SD* = 0.73), the KMO was 0.87, and Bartlett’s test was significant, χ^2^(36) = 878.34, *p* < 0.001. Both the scree plot and Kaiser’s K > 1 criterion suggested a one-factor solution. Parallel Analysis (9 variables, 342 participants, 1000 replications) confirmed this decision: the first observed eigenvalue (3.91) exceeded the 95th percentile random eigenvalue (1.33), while the second observed eigenvalue (0.95) did not. The single extracted factor had an eigenvalue of 3.91 and explained 43.48% of the total variance.

All nine facets demonstrated adequate factor loadings (range: 0.56 to 0.74; all loadings > 0.40) on the extracted factor. Communalities ranged from 0.31 to 0.55 (*M* = 0.44). This finding was consistent with the total sample and girls’ results, supporting the unifactorial structure of trait EI in younger children.

**Figure 3:**
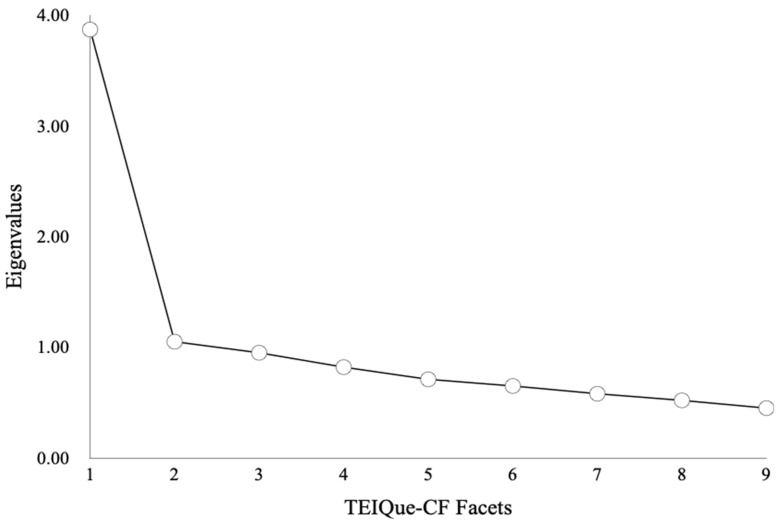
Scree Plot for PCA of the Nine Facets of the TEIQue-CF for the Older Children (*n* = 331).

For older children (Years 5–6; *n* = 331; *M* age = 10.26, *SD* = 0.70), the results paralleled those observed in boys. The KMO was 0.86, and Bartlett’s test was significant, χ^2^(36) = 861.99, *p* < 0.001. To determine the most appropriate factor solution, multiple criteria were employed. The scree plot (see Figure 3) suggested a two-factor solution. Applying Kaiser’s K > 1 criterion, two factors were extracted and accounted for 54.67% of the total variance. However, Parallel Analysis (9 variables, 331 participants, 1000 replications) again indicated only one factor should be retained: while the first observed eigenvalue (3.87) exceeded the 95th percentile random eigenvalue (1.34), the second observed eigenvalue (1.05) did not, suggesting the two-factor solution may be artificial.

Despite the PA recommendation for a one-factor solution, the two-factor structure was examined due to theoretical interest in potential emerging differentiation. The two-factor solution for older children was rotated using Direct Oblimin rotation with Kaiser Normalization. As shown in Table 5, the first factor (eigenvalue = 3.87, 43.01% variance) comprised six facets: Self-esteem (0.76), Adaptability (0.79), Peer relations (0.76), Affective disposition (0.59), Emotion expression (0.56), and Emotion perception (0.35). The second factor (eigenvalue = 1.05, 11.65% variance) comprised three facets: Impulse control (0.86), Self-motivation (0.70), and Emotion regulation (0.60). The factors were again labeled Socioemotionality and Emotion control. The correlation between the two factors was 0.41.

### 2.3. Discussion

We found a clear and developmentally coherent picture. Across the nine TEIQue-CF facets, a single, broad trait EI dimension best accounted for responses at the facet level, with PA supporting a unifactorial solution in the full sample. Subgroup checks converged on the same conclusion for girls and for younger pupils (Years 3 to 4). Although the scree and K > 1 rules suggested two factors for boys and for older pupils (Years 5 to 6), PA did not retain a second factor. When we nevertheless inspected the exploratory two-factor pattern, facets clustered in an interpretable way: Self-esteem, Peer relations, Adaptability, Affective disposition, Emotion expression, and Emotion perception on one factor (“Socioemotionality”), and Impulse control, Emotion regulation, and Self-motivation on a second (“Emotion control”). However, these splits should be viewed as tentative given the PA results. Taken together, the Study 1 evidence indicates a largely undifferentiated trait EI structure in middle childhood, with hints of emerging differentiation in some subgroups that warrant replication.

Measurement characteristics supported that interpretation. Facet reliabilities were acceptable for brief subscales overall, with somewhat higher internal consistency in Years 5 to 6 than in Years 3 to 4; a few facets (e.g., Adaptability, Emotion expression, Emotion perception) fell just below conventional thresholds in the total sample and more often so among younger pupils, a common consequence of short scales that sample broad content in children. Score distributions were mostly reasonable but showed the expected negative skew on facets such as Self-motivation and Self-esteem, consistent with classroom administration and positively biased responding in this age range, which are factors that can inflate a general factor and dampen facet differentiation. Overall, these psychometric patterns are consistent with children’s still-forming self-concepts and support using the global TEIQue-CF score in this age band while treating fine-grained facet contrasts with caution, especially in younger or less mature children. While a two-factor solution provided a conceptually interpretable and parsimonious description of the item covariation in this sample, the second factor was not clearly retained according to parallel analysis criteria. Accordingly, this structure should be viewed as preliminary and descriptive rather than confirmatory, pending replication in independent samples.

## 3. Study 2

Study 2 evaluated the observed component structure of the TEIQue-CSF at the end of primary school, using a large, single-year cohort. Whereas Study 1 mapped the dimensionality of the full 75-item TEIQue-CF across Years 3–6, here we test whether the short form, designed to yield a global trait EI score while still sampling all nine child facets but without providing facet-level measurement, shows a similar structure at parcel level. Building on Study 1 and prior full-form evidence, we expected a one- or two-factor solution to provide the best account of the parcel correlations in this age group.

Beyond simple replication, Study 2 examined whether the qualitative pattern observed in Study 1 reappeared when trait EI was sampled with the 36-item TEIQue-CSF. Specifically, we asked whether a single factor sufficed or whether an oblique two-factor solution was required. Because the short form was built to yield a global score while retaining coverage of the nine child facets, we analysed parcels rather than items. We expected that either a single factor would be retained or two factors would form an interpretable oblique structure aligned with the Study 1 factors. In a two-factor solution, Socioemotionality would be defined by Self-esteem, Peer relations, Adaptability, Affective disposition, Emotion expression, and Emotion perception, whereas Emotion control would be defined by Impulse control, Emotion regulation, and Self-motivation.

### 3.1. Method

#### 3.1.1. Participants

Participants were 1582 Year 6 pupils (ages 11–12; 803 boys, 693 girls, 86 unreported) drawn from 44 schools in the Borough of Blackburn with Darwen. Self-reported ethnic composition was 60.5% British White, 17.9% British Indian, 15.7% British Pakistani, 2.4% Other.

#### 3.1.2. Measure

*Trait Emotional Intelligence Questionnaire–Child Short Form (TEIQue-CSF).* The short form was constructed from the TEIQue-CF to provide an efficient index of children’s trait EI with full sampling of the nine child facets: Adaptability, Affective disposition, Emotion expression, Emotion perception, Emotion regulation, Impulse control, Peer relations, Self-esteem, and Self-motivation. Item selection prioritised internal consistency and facet-level intercorrelations in the development sample of the full form, with 3–4 items representing each facet (for a total of 36 items). This procedure ensured that the selected items provided comprehensive coverage of all nine facets of children’s sampling domain of trait EI. As in the full form, items were first-person statements rated on a five-point scale and written at a primary-appropriate reading level. Piloting established comprehension and completion time, with iterative wording refinements to ensure suitability for ages 8–12 years.

#### 3.1.3. Procedure

The administration procedures mirrored those employed in Study 1. Schools were approached with detailed information regarding the study’s aims and procedures, and participating schools provided institutional consent. Questionnaires were administered collectively in classrooms by school staff following a standardized protocol. Prior to administration, pupils were informed that participation was voluntary, that they could omit any item or withdraw at any time without penalty, and that all responses would be treated confidentially. All procedures adhered to the ethical standards of the British Psychological Society and the Declaration of Helsinki. The study protocol was reviewed and approved by the institutional Research Ethics Committee.

#### 3.1.4. Statistical Analysis

Because the TEIQue-CSF is intended to support a single global score rather than facet-level interpretation, structural analyses were conducted at the parcel level. As per the scoring instructions of the TEIQue-CSF, some items were reverse-scored prior to computing parcels using mean scores. Nine parcels were formed by aggregating the items defining each facet: Adaptability, Affective disposition, Emotion expression, Emotion perception, Emotion regulation, Impulse control, Peer relations, Self-esteem, and Self-motivation.

Analyses targeted factor retention and interpretability at the parcel level. We first examined parcel score distributions and computed internal consistency for the global TEIQue-CSF score. Sampling adequacy and the suitability of the correlation matrix for factor analysis were evaluated via the KMO measure and Bartlett’s test of sphericity. Parcels were then analyzed using PCA followed by Direct Oblimin rotation with Kaiser Normalization, since parcels were expected to intercorrelate. Multiple criteria guided factor retention, including visual inspection of the scree plot, Kaiser’s K > 1 criterion, and PA performed as in Study 1 with 1000 Monte Carlo replications using the 95th-percentile criterion at α = 0.05. Finally, stability checks were conducted by repeating all structure-finding procedures separately for boys and girls, with resulting patterns compared to Study 1 findings to determine whether any multi-factor solutions demonstrated replication across subgroups.

### 3.2. Results

#### 3.2.1. Score Distributions and Internal Consistency

We analysed parcels formed from the nine TEIQue-CSF facets. Parcel distributions showed acceptable shape characteristics for PCA. Across parcels, skewness ranged from −0.53 to −0.02, and kurtosis ranged from −0.20 to 0.26, all within the a priori acceptability guideline of |2| for skewness and |7| for kurtosis. Visual inspection of histograms revealed no extreme outliers, and no transformations were applied.

K-S tests indicated that several individual parcels deviated from normality (K-S values = 0.07–0.10, *p*s < 0.05), which is common in child samples with short parcels. In contrast, the global TEIQue-CSF score did not depart from normality (K-S(1582) = 0.03, *p* = 0.195). Given the parcel distributions and the robustness of PCA to mild non-normality, all parcels were retained for subsequent analyses. There was evidence of systematic negative skew on Self-motivation and Affective disposition (<−0.5), consistent with the classroom testing context and with positively keyed content in primary-age respondents. This pattern is noted here to aid interpretation but does not affect our retention decisions.

Internal consistency for the global TEIQue-CSF score was good (α = 0.82). Parcel-level reliabilities ranged from 0.32 to 0.63 (Adaptability = 0.45, Emotion expression = 0.41, Emotion perception = 0.32, Self-motivation = 0.44, Self-esteem = 0.46, Impulse control = 0.49, Peer relations = 0.37, Emotion regulation = 0.48, Affective disposition = 0.63). Modest coefficients at the parcel level are expected given the very small number of items per parcel (four items each) in child assessments.

Interparcel correlations were positive and in the expected range, supporting the use of an oblique rotation in PCA. The median interparcel correlation was 0.33 (range = 0.10–0.52). Highest was Self-esteem and Peer relations (0.52), and the lowest was Adaptability and Impulse control (0.10).

#### 3.2.2. Factor Analysis

##### Total Sample

**Figure 4:**
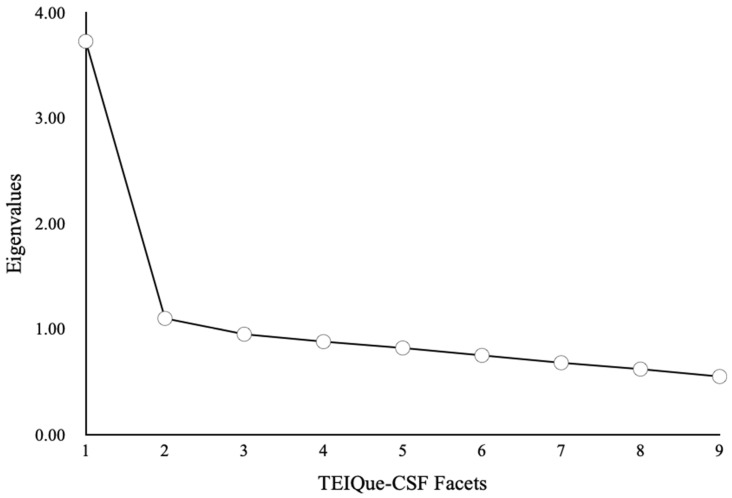
Scree Plot for PCA of the Nine Facets of the TEIQue-CSF for the Total Sample (*N* = 1582).

In the full Year 6 sample (*N* = 1582), the KMO was 0.86, and Bartlett’s test was significant, χ^2^(36) = 3783.13, *p* < 0.001, indicating that the correlation matrix was appropriate for factor analysis. The scree plot and Kaiser’s K > 1 criterion both indicated a two-factor solution, which PA corroborated, albeit very marginally, as non-trivial. The first two observed eigenvalues were 3.73 and 1.10 (see Figure 4), accounting for 41.5% and 12.3% of the variance, respectively (total = 53.7%).

The rotated pattern reproduced the interpretable configuration anticipated from Study 1: a broad *Socioemotionality* factor defined by Adaptability (0.79), Peer relations (0.75), Self-esteem (0.70), Emotion expression (0.55), Affective disposition (0.52), and Emotion perception (0.51), and an *Emotion Control* factor defined by Impulse control (0.88), Emotion regulation (0.72), and Self-motivation (0.65). The two factors were moderately correlated at *r* = 0.39. Loadings are summarised in Table 6 below.

#### 3.2.3. Subgroup Analyses by Gender

##### Gender Differences

The data were subsequently factor analysed separately by gender. For girls (*n* = 693), the KMO was 0.85, and Bartlett’s test was significant, χ^2^(36) = 1670.81, *p* < 0.001. Kaiser’s K > 1 criterion marginally suggested a two-factor solution, with the second eigenvalue (1.10) just exceeding unity. However, Parallel Analysis (9 variables, 693 participants, 1000 replications) indicated only one factor should be retained: the first observed eigenvalue (3.73) exceeded the 95th percentile random eigenvalue (1.23), while the second observed eigenvalue (1.10) did not exceed its corresponding random eigenvalue (1.16).

Despite the PA recommendation for a one-factor solution, the two-factor structure was examined due to theoretical interest in potential emerging differentiation. The two-factor solution for girls was rotated using Direct Oblimin rotation with Kaiser Normalization. As shown in Table 7, the first factor (eigenvalue = 3.73, 41.44% variance) comprised six facets: Peer relations (0.81), Emotion expression (0.71), Adaptability (0.70), Self-esteem (0.61), Affective disposition (0.50), and Emotion perception (0.49). The second factor (eigenvalue = 1.10, 12.24% variance) comprised three facets: Impulse control (0.86), Self-motivation (0.71), and Emotion regulation (0.71). The first factor was labelled *Socioemotionality*, and the second factor was labelled *Emotion control*. The correlation between the two factors was 0.42.

For boys (*n* = 803), the results were comparable. The KMO was 0.86, and Bartlett’s test was significant, χ^2^(36) = 1881.58, *p* < 0.001. To determine the most appropriate factor solution, multiple criteria were employed. The scree plot suggested a two-factor solution. Applying Kaiser’s K > 1 criterion, two factors were extracted, accounting for 53.29% of the total variance. However, Parallel Analysis (9 variables, 803 participants, 1000 replications) indicated only one factor should be retained: while the first observed eigenvalue (3.71) exceeded the 95th percentile random eigenvalue (1.21), the second observed eigenvalue (1.09) did not exceed the corresponding random eigenvalue (1.14), suggesting the two-factor solution may be artifactual.

Despite the PA recommendation for a one-factor solution, the two-factor structure was examined due to theoretical interest in potential emerging differentiation. The two-factor solution for boys was rotated using Direct Oblimin rotation with Kaiser Normalization. As shown in Table 8, the first factor (eigenvalue = 3.71, 41.21% variance) comprised six facets: Adaptability (0.81), Peer relations (0.73), Self-esteem (0.72), Affective disposition (0.56), Emotion perception (0.51), and Emotion expression (0.46). The second factor (eigenvalue = 1.09, 12.08% variance) comprised three facets: Impulse control (0.89), Emotion regulation (0.67), and Self-motivation (0.59). The first factor was labelled *Socioemotionality*, and the second factor was labelled *Emotion control*. The correlation between the two factors was 0.35.

In summary, the two-factor structure observed in the total sample was replicated across both gender subgroups. Although Parallel Analysis recommended a one-factor solution for both girls and boys, the two-factor solutions when extracted showed consistent factor composition: *Socioemotionality* (comprising Adaptability, Peer relations, Self-esteem, Emotion expression, Affective disposition, and Emotion perception) and *Emotion control* (comprising Impulse control, Emotion regulation, and Self-motivation). The factor correlations were moderate in both subgroups (*r* = 0.42 for girls; *r* = 0.35 for boys), consistent with the total sample correlation of *r* = 0.39. Minor variations in factor loadings and facet ordering were observed between genders, but the overall pattern indicates structural similarity of the TEIQue-CSF factor structure across gender in this age group. Parallel analysis retained two factors in the total Study 2 sample, although this support was marginal; in contrast, parallel analysis recommended a one-factor solution in both gender subgroups, so the bifactorial subgroup solutions should be interpreted descriptively. Nonetheless, the two-factor pattern was structurally coherent across groups even when the retention criterion was marginal.

### 3.3. Discussion

Study 2 extends the findings from Study 1 by demonstrating that the structure of trait EI becomes more clearly differentiated as children approach preadolescence. Whereas the younger and more heterogeneous sample in Study 1 yielded a predominantly unifactorial structure, the Year 6 cohort examined here showed evidence consistent with a two-factor pattern. These findings are in line with theoretical accounts of self-concept formation in late childhood.

The emergence of distinct Socioemotionality and Emotion control factors at ages 11–12 has a plausible developmental basis. By this stage, children have accumulated years of feedback from teachers, parents, and peers that helps them differentiate between how they relate to others emotionally and how they manage their own internal states. Descriptions of middle-childhood socioemotional development emphasise growing awareness of emotional expression, emerging understanding of more complex emotions (e.g., embarrassment, pride), and increasing ability to suppress negative reactions and regulate emotion in socially expected ways, supporting our findings (e.g., Erasmus et al., 2022).

Socioemotionality as a factor captures self-perceptions about interpersonal functioning and emotional experience, while Emotion control reflects awareness of one’s capacity for self-regulation. This separation mirrors the increasing emphasis placed on behavioural self-control as children transition toward secondary school, where expectations for emotional maturity and impulse management become more explicit.

Factor structure replicated across gender subgroups in a noteworthy manner. Although PA was conservative in both cases, the consistent composition of the two factors suggests that the distinction between Socioemotionality and Emotion control is not an artefact of a particular subsample. Structural coherence strengthens the case for interpreting these observed component structures, as meaningful aspects of how children at the end of primary school understand their emotional selves.

When considered alongside Study 1, the present findings suggest a developmental trajectory in trait EI structure. By Year 6, the tentative differentiation observed in older pupils and boys in Study 1 appears to consolidate, producing a more stable two-factor solution. Developmental research supports such a pattern, indicating that self-perceptions become increasingly differentiated and domain-specific as children mature. Across all analyses, the moderate correlation between factors confirms that Socioemotionality and Emotion control remain related constructs, united under the trait EI umbrella, while nonetheless capturing distinguishable facets of children’s affective personality.

The measurement properties of the TEIQue-CSF support its use as a brief, age-appropriate index of trait EI in late childhood. Modest reliabilities at the parcel level are a predictable consequence of the short-form design and do not undermine the structural findings, particularly given the strong internal consistency of the global composite. Consistent with this intended use, the TEIQue-CSF is explicitly framed as yielding a single global trait EI score for children and has been reported to provide reliable coverage of child trait EI (8–12 years), with good internal consistency in primary-school samples (Agnoli et al., 2023; Stassart et al., 2017).

Negative skew appearing on certain parcels reflects the well-documented tendency for children to report favourably on their own characteristics, especially in classroom settings. More generally, studies relying on children’s self-reports caution that responding may be influenced by perceived social desirability (Stassart et al., 2017), which would be expected to shift scores upward and is consistent with the ceiling-leaning (negatively skewed) distributions observed here when higher scores reflect more desirable attributes. It is to be noted most strongly that the TEIQue-CSF is not intended for facet-level scoring, and such use is not recommended. Parcels were used in this case solely for the purposes of exploring underlying factor structure.

In sum, Study 2 demonstrates that the largely undifferentiated trait EI structure observed in middle childhood gives way to a two-factor organisation by the end of primary school. Separating Socioemotionality from Emotion control corresponds to developmental gains in metacognitive awareness, emotion vocabulary, and self-regulatory capacity that characterise the transition toward adolescence. Together with Study 1, these findings map a structural trajectory that has implications for how trait EI should be scored and interpreted across the primary school years.

## 4. General Discussion

The present research examined the factorial structure of trait emotional intelligence (EI) in childhood through the development and validation of the TEIQue-CF and TEIQue-CSF across two studies spanning middle and late childhood. Our findings reveal a developmentally sensitive factor structure: younger children demonstrated a predominantly unifactorial conceptualization of trait EI, while older children showed evidence of differentiation into two distinct but related factors: Socioemotionality and Emotion control. These results illuminate how children’s understanding of their emotional self becomes increasingly refined and multidimensional with age, providing important insights into both the construct of trait EI in childhood and the broader developmental trajectory of emotional self-concept.

### 4.1. Developmental Differentiation of Trait EI

The observed shift from a unifactorial to a two-factor structure across childhood stages aligns with fundamental principles of self-concept development. Younger children appear to hold a more holistic, undifferentiated view of their emotional functioning, consistent with research demonstrating that children’s self-descriptions become progressively more differentiated, abstract, and psychologically nuanced throughout middle childhood and adolescence (e.g., Marsh & Ayotte, 2003). Evidence from the unifactorial solution in Study 1 suggests that younger children conceive of their emotional competencies as a general, unified positive attribute rather than distinguishing between distinct emotional domains. Such a global self-perception likely reflects both cognitive limitations in making fine-grained distinctions between related psychological constructs and the characteristically optimistic self-evaluations typical of younger children (Harter, 2012). However, this pattern should be interpreted as tentative rather than a definite developmental transition as the observed differentiation may reflect both genuine developmental processes and methodological effects associated with age, differences in instrument form (full vs. short) and level of analysis (facet scores versus parcels). Future longitudinal research can establish whether the observed pattern reflects underlying structural change during the primary school years.

As children mature into late childhood, they develop the cognitive sophistication and metacognitive capacity to differentiate between how they experience and express emotions socially (Socioemotionality) and how they manage and regulate emotional responses (Emotion control). Developmental progression during this stage mirrors broader transformations in the self-system, including advances in abstract reasoning, heightened self-reflection, and increasing recognition of trait-like consistencies in behavior across contexts (Barenboim, 1981; Crone & van Drunen, 2024). The clearest evidence for this two-factor structure emerged in the Year 6 sample (ages 11–12), although Study 1 suggested only tentative signs of differentiation in older children. This developmental period coincides with significant advances in emotion regulation, including the increasing use of cognitive reappraisal strategies and other mentalistic approaches to emotional management (Garnefski et al., 2007; Willner et al., 2022). However, the use of parcel-level analysis in Study 2 may have facilitated the emergence of more clearly defined factor structures; therefore, the observed differentiation may reflect analytic characteristics of the parceling approach in addition to developmental processes.

### 4.2. Cross-Cultural Consistency and Validation

Our findings converge remarkably with validation studies of the TEIQue-CF across diverse cultural contexts. A two-factor solution comprising Socioemotionality and Emotion control has been replicated in Serbian, Turkish, and French-speaking samples (Banjac et al., 2016; Beyazit et al., 2020; Stassart et al., 2017), while the unifactorial structure for younger children has been observed in Italian and Serbian populations (Banjac et al., 2016; Russo et al., 2011). From a theoretical perspective, cross-cultural consistency suggests that differentiation in trait EI development may reflect universal cognitive-developmental mechanisms rather than patterns driven by culture-specific socialization. The replication of these factorial structures across linguistic and cultural boundaries strengthens the theoretical validity of trait EI as a developmental construct and supports the cross-cultural applicability of the TEIQue child forms.

### 4.3. Theoretical Implications and Underlying Mechanisms

A developmental shift in factorial structure is theoretically consequential for understanding trait EI in childhood. First, it suggests that trait EI should not be conceptualized as a static construct but rather as one that undergoes qualitative reorganization as children mature. Greater differentiation likely arises from the convergence of several developmental processes, including maturation of prefrontal neural systems supporting emotion regulation (van der Cruijsen et al., 2023), growth in abstract cognitive and psychological understanding, expansion of social experiences that demand advanced emotional skills, and increasing metacognitive awareness of personal emotional functioning.

Second, the two-factor solution that emerges in late childhood appears to capture a meaningful distinction between emotional experience/expression and emotional management. This separation may reflect children’s developing recognition that feeling and relating emotionally to others (Socioemotionality) involves different competencies than controlling impulses and regulating emotional states (Emotion control). Such differentiation likely serves adaptive functions as older children face increasingly complex social demands, peer evaluation pressures, and adult expectations of behavioral self-control.

Results from the observed factor structure shed light on the relationship between trait EI and personality development more broadly. The increasing complexity and differentiation in children’s emotional self-perceptions mirrors the refinement of personality structure throughout childhood and adolescence (Shiner et al., 2021). As children’s social worlds expand and they encounter diverse situational demands, they must develop more nuanced understandings of their emotional tendencies to navigate these contexts effectively.

### 4.4. Methodological Considerations

Early scree plot analyses pointed to possible two-factor solutions among younger children and boys; however, results from parallel analysis with a more empirically grounded extraction method suggested that these factors may be subject to artificial influences. A key methodological lesson concerns the value of employing multiple factor extraction techniques, particularly when working with developmental populations in which factor structures may be less clearly defined. Convergence between the full and short forms of the TEIQue-CF in revealing similar developmental patterns enhances confidence in the findings.

The lower internal consistency observed for some facets in younger children, consistent with previous studies (Banjac et al., 2016; Russo et al., 2011), likely reflects the less differentiated nature of younger children’s emotional self-concept rather than fundamental measurement problems. As children’s understanding of emotional constructs becomes more refined, the coherence of their responses to items assessing specific facets naturally increases.

### 4.5. Practical Applications

Our findings have implications for assessment and intervention in educational and clinical contexts. Practitioners working with younger children should recognize that global trait EI scores may be more meaningful and psychometrically sound than subscale scores, given the unifactorial structure. For older children approaching adolescence, differentiated assessment of Socioemotionality and Emotion control becomes appropriate and may provide more actionable information for targeted interventions. Educational programs aimed at enhancing emotional competencies might benefit from emphasizing integrated emotional development in younger children while introducing more differentiated skill-building focused on specific domains (e.g., explicit emotion regulation strategies) as children mature.

### 4.6. Limitations and Future Directions

Several limitations warrant consideration. Specifically, across some trait EI facets, the data were negatively skewed. Children’s responses can be positively biased (e.g., Thomaes et al., 2017), and we considered these results normal. For example, the non-normal distribution of the Self-motivation facet is unsurprising, given that the facet comprised items related to schoolwork (e.g., “I try to do my homework as well as I really can.”). The classroom context and the presence of the teacher might also have influenced children’s responses.

While our sample size was adequate for factor analysis, replication with larger samples across diverse age ranges would strengthen confidence in the observed developmental patterns and allow for more fine-grained examination of when exactly the structural shift occurs. The cross-sectional design prevents us from drawing definitive conclusions about intra-individual developmental trajectories; longitudinal research following children from middle childhood through adolescence would provide more definitive evidence of how trait EI structure evolves within individuals over time. Furthermore, parceling was employed consistent with the focus on higher-order structure rather than facet-level interpretation. However, this approach can artificially enhance factor clarity and should be considered when contrasting developmental patterns with Study 1’s facet-level results.

In addition, formal tests of factorial invariance across gender were not conducted; replication using multi-group confirmatory factor analytic approaches would provide stronger evidence of structural equivalence.

Future research should continue to scrutinize the cross-cultural validity and measurement invariance of the TEIQue child forms to establish whether the developmental patterns we observed generalize across diverse cultural contexts or reflect culture-specific developmental processes. Investigation of the incremental validity of the TEIQue-CF and TEIQue-CSF beyond established personality dimensions would further establish their unique contribution to predicting important developmental outcomes such as academic achievement, social adjustment, and psychological well-being. For example, TEIQue-CF scores have been linked to psychosocial adjustment in 8- to 12-year-olds while accounting for social-emotional problems (Piqueras et al., 2019), and trait EI has also been associated with children’s psychological well-being after general intelligence (IQ) is considered (Pauletto et al., 2021).

A further limitation pertains to the alpha values. Although modest alphas are common in very short parcels with child samples, values in this range indicate potential instability in the derived solutions and warrant replication with larger or more reliable indicators.

Furthermore, research investigating potential moderators of factor structure development, such as cognitive ability, social experience diversity, or educational environment would deepen our understanding of the mechanisms driving trait EI differentiation. Consistent with this possibility, studies combining the TEIQue-CF with measures of fluid intelligence show that cognitive ability and trait EI can make age- and group-dependent contributions to affective decision-making in childhood and early adolescence (Li et al., 2017, 2020).

## 5. Conclusions

Our research suggests that trait EI in childhood is characterized by a developmentally sensitive factor structure that shows increasing differentiation with age in the organisation of self-report data. Younger children’s responses were primarily structured around a more unified, global dimension of emotional functioning, whereas older children’s self-reports showed a more differentiated configuration reflecting socioemotional and emotion control domains. Patterns of this kind are consistent with broader growth in cognitive and emotional understanding and self-regulatory systems during childhood (Pons et al., 2004; Roebers, 2017).

The TEIQue-CF and TEIQue-CSF provide psychometrically sound, developmentally appropriate tools for assessing trait EI in childhood. The observed factorial configurations align with age-related differences in the statistical organisation of children’s responses. Collectively, these findings contribute to our theoretical understanding of trait EI as a developmental construct and inform its assessment across childhood, while remaining subject to further confirmation through longitudinal and confirmatory analytic approaches. Recommendations regarding the prioritisation of global versus differentiated scoring, while potentially informative for educational and clinical contexts, should be regarded as provisional and contingent upon future validation.

## Figures and Tables

**Table 1:** The Sampling Domain of Trait Emotional Intelligence in Children.

Facet	Brief Description	Example Item
Adaptability	Children’s perceptions of how well they adapt to new situations and people.	“I find it hard to get used to a new school year.”
Affective disposition	Children’s perceptions of the frequency and intensity with which they experience emotions.	“I’m a very happy kid.”
Emotion expression	Children’s perceptions of how effectively they can express their emotions.	“I always find the words to show how I feel.”
Emotion perception	Children’s perceptions of how accurately they identify their own and others’ emotions.	“It’s easy for me to understand how I feel.”
Emotion regulation	Children’s perceptions of how well they can control their emotions.	“I can control my anger.”
Impulse control	Children’s perceptions of how effectively they can control themselves.	“I don’t like waiting to get what I want.”
Peer relations	Children’s perceptions of the quality of their relationships with their classmates.	“I listen to other children’s problems.”
Self-esteem	Children’s perceptions of their self-worth.	“I feel great about myself.”
Self-motivation	Children’s perceptions of their drive and motivation.	“I always try to become better at school.”

**Table 2:** Internal Consistencies, Correlations, Means, Standard Deviations, and Number of Items for the Nine TEIQue-CF Facets in the Total Sample (*N* = 720).

Facet	Skewness	Kurtosis	α	1	2	3	4	5	6	7	8	9
1. Adaptability	−0.15	−0.26	0.59	-								
2. Affective disposition	−0.67	0.39	0.77	0.31	-							
3. Emotion expression	−0.12	0.00	0.59	0.28	0.33	-						
4. Emotion perception	−0.18	−0.06	0.57	0.32	0.35	0.36	-					
5. Emotion regulation	−0.22	−0.08	0.61	0.25	0.44	0.36	0.37	-				
6. Impulse control	−0.21	0.17	0.65	0.17	0.39	0.31	0.31	0.50	-			
7. Peer relations	−0.52	0.28	0.63	0.45	0.46	0.42	0.40	0.46	0.32	-		
8. Self-esteem	−0.55	0.35	0.70	0.25	0.48	0.41	0.36	0.34	0.23	0.53	-	
9. Self-motivation	−0.53	0.01	0.62	0.30	0.33	0.27	0.38	0.40	0.40	0.41	0.35	-
*Mean*	-	-	-	3.58	3.66	3.24	3.70	3.43	3.30	3.77	3.69	4.11
*SD*	-	-	-	0.67	0.78	0.63	0.59	0.67	0.70	0.53	0.70	0.59
*N items*	-	-	-	8	8	8	8	8	8	12	7	8

**Table 3:** Internal Consistencies for the TEIQue-CF and the Nine Facets for Pupils from Years 3–4 and Years 5–6.

Facet	Number of Items	Years 3–4 *n* = 342	Years 5–6 *n* = 331
TEIQue-CF	75	0.89	0.91
1. Adaptability	8	0.51	0.67
2. Affective disposition	8	0.74	0.79
3. Emotion expression	8	0.55	0.62
4. Emotion perception	8	0.54	0.61
5. Emotion regulation	8	0.57	0.63
6. Impulse control	8	0.61	0.69
7. Peer relations	12	0.61	0.65
8. Self-esteem	7	0.69	0.72
9. Self-motivation	8	0.61	0.62

**Table 4:** Factor Loadings of the TEIQue-CF in the Boys’ Sample (*n* = 343).

	Factor Loadings	
Facet	Socioemotionality	Emotion Control
1. Self-esteem	0.72	
2. Peer relations	0.79	
3. Adaptability	0.75	
4. Emotion expression	0.58	
5. Emotion perception	0.50	
6. Affective disposition	0.46	
7. Impulse control		0.90
8. Emotion regulation		0.71
9. Self-motivation		0.52
Eigenvalues	3.74	1.04
% of variance explained	41.56	11.50

**Table 5:** Factor Loadings of the TEIQue-CF in the Older Children’s Sample (*n* = 331).

	Factor Loadings	
Facet	Socioemotionality	Emotion Control
1. Self-esteem	0.76	
2. Adaptability	0.79	
3. Peer relations	0.76	
4. Affective disposition	0.59	
5. Emotion expression	0.56	
6. Emotion perception	0.35	
7. Impulse control		0.86
8. Self-motivation		0.70
9. Emotion regulation		0.60
Eigenvalues	3.87	1.05
% of variance explained	43.01	11.65

**Table 6:** Factor Loadings of the TEIQue-CSF in the Total Sample (*N* = 1582).

	Factor Loadings	
Facet	Socioemotionality	Emotion Control
1. Adaptability	0.79	
2. Peer relations	0.75	
3. Self-esteem	0.70	
4. Emotion expression	0.55	
5. Affective disposition	0.52	
6. Emotion perception	0.51	
7. Impulse control		0.88
8. Emotion regulation		0.72
9. Self-motivation		0.65
Eigenvalues	3.73	1.10
% of variance explained	41.5	12.3

Note. Direct Oblimin rotation with Kaiser Normalization. Factor correlation *r* = 0.39. Only primary loadings ≥ |0.40| are displayed.

**Table 7:** Factor Loadings of the TEIQue-CSF in the Girls’ Sample (*n* = 693).

	Factor Loadings	
Facet	Socioemotionality	Emotion Control
1. Peer relations	0.81	
2. Emotion expression	0.71	
3. Adaptability	0.70	
4. Self-esteem	0.61	
5. Affective disposition	0.50	
6. Emotion perception	0.49	
7. Impulse control		0.86
8. Self-motivation		0.71
9. Emotion regulation		0.71
Eigenvalues	3.73	1.10
% of variance explained	41.44	12.24

*Note*. Direct Oblimin rotation with Kaiser Normalization. Factor correlation *r* = 0.42. Only primary loadings ≥ |0.40| are displayed.

**Table 8:** Factor Loadings of the TEIQue-CSF in the Boys’ Sample (*n* = 803).

	Factor Loadings	
Facet	Socioemotionality	Emotion Control
1. Adaptability	0.81	
2. Peer relations	0.73	
3. Self-esteem	0.72	
4. Affective disposition	0.56	
5. Emotion perception	0.51	
6. Emotion expression	0.46	
7. Impulse control		0.89
8. Emotion regulation		0.67
9. Self-motivation		0.59
Eigenvalues	3.71	1.09
% of variance explained	41.21	12.08

*Note*. Direct Oblimin rotation with Kaiser Normalization. Factor correlation *r* = 0.35. Only primary loadings ≥ |0.40| are displayed.

## Data Availability

The data presented in this study are available on request from the corresponding author.
